# Changes in 24 h Rhythmicity of Spontaneous Locomotor Activity in the Triple Transgenic Mouse for Alzheimer’s Disease (3xTg-AD) in a Jet Lag Protocol: Correlations with Retinal Sensitivity

**DOI:** 10.5334/jcr.214

**Published:** 2021-05-27

**Authors:** Irma Angélica González-Luna, Cinthia Juárez-Tapia, Azucena Aguilar-Vázquez, Edith Arnold, Sofia Díaz-Cintra, Manuel Miranda-Anaya, Mauricio Díaz-Muñoz

**Affiliations:** 1 Departamento de Neurobiología Celular y Molecular, Instituto de Neurobiología, Universidad Nacional Autónoma de México, Querétaro, México; 2 Unidad Multidisciplinaria de Docencia e Investigación (UMDI), Facultad de Ciencias, Universidad Nacional Autónoma de México, Querétaro, México; 3 Departamento de Neurobiología del Desarrollo y Neurofisiología, Universidad Nacional Autónoma de México, Querétaro, México

**Keywords:** Alzheimer’s disease, circadian rhythms, jet lag, locomotor activity, retina, 3xTg-AD

## Abstract

The progression of amyloid plaques and neurofibrillary tangles in different brain areas is associated with the effects of Alzheimer’s disease (AD). In addition to cognitive impairment, circadian alterations in locomotor activity have also been detected, but they have not been characterized in a jet lag protocol. Therefore, the present study aimed to compare 3xTg-AD and non-transgenic mice in changes of 24 h cycles of spontaneous locomotor activity in a jet lag protocol, in an environment without a running wheel, at 3 different states of neuronal damage: early, intermediate and advanced (3, 8 and 13 months, respectively). The 3xTg-AD mice at 3 months presented differences in phase angle and acrophase, and differentially increased activity after advances more than after delays. At 13 months, a shortening of the free-running period in constant darkness was also noted. 3xTg-AD mice showed a significant increase (123%) in global activity at 8 to 13 months and in nighttime activity (153%) at 13 months. In the advance protocol (ADV), 3xTg-AD mice displayed a significant increase in global activity (171%) at 8 and 13 months. The differences in masking effect were evident at 8 months. To assess a possible retinal dysfunction that could interfere with photic entrainment as part of the neurodegenerative process, we compared electroretinogram recordings. The results showed early deterioration in the retinal response to light flashes in mesopic conditions, observed in the B-wave latency and amplitude. Thus, our study presents new behavioral and pathological characteristics of 3xTg-AD mice and reveals the usefulness of non-invasive tools in early diagnosis.

## Introduction

It is currently estimated that 44 million people worldwide have dementia. Of these, 60–80% are due to Alzheimer’s disease (AD) [1,2]. Among the non-cognitive symptoms of AD are circadian rhythm disturbances, which affect more than 80% of individuals over the age of 65 [3]. These disturbances include disruptions in the rest-activity cycle such as sleep-wake fragmentation in the form of frequent daytime napping and nighttime awakenings, delayed phase of sleep onset, decreased amplitude of melatonin synthesis rate, decreased regulation of body temperature [4,5,6], changes in energy metabolism [7], exacerbated agitation, and increased locomotor activity with anxiety and delirium during the evening (a condition known as “sundowning”) [3,8,9].

The main circadian clock in mammals is the suprachiasmatic nucleus (SCN). Its photic entrainment (effect of light on the regulation of internal biological rhythms) starts in the intrinsically photosensitive retinal ganglion cells (ipRGCs) that send information through the retinohypothalamic tract (RHT) and release glutamate and PACAP to neurons and glia within the SCN [10]. This information influences clock genes, which control all aspects of physiological activities and are involved in generating and maintaining circadian rhythms by means of two transcriptional activation/repression loops: a positive one controlled by *Clock* and *Bmal1* genes and a negative one controlled by *Per* and *Cry*. The chemical and electrical coupling between SCN neurons influences neuroendocrine and behavioral outputs that affect virtually all tissues within the organism (peripheral oscillators). However, after altered photoperiods (e.g., extended workdays) [11] or transmeridian travel [12], circadian resynchronization occurs between the SCN, the external light-dark cycle and the peripheral oscillators. This process of resynchronization, associated with various metabolic, physiological and behavioral changes, and with a deficit in cognitive processes such as attention, memory and reaction times, is known as jet lag [13,14,15,16].

The study of AD requires biological models, and particularly useful is the availability of transgenic animals. Many genetically manipulated mice exist to study AD; they encompass familial, sporadic, and early- and late-onset forms of this malady [17]. In this context, one of the most employed models is the triple transgenic mouse for Alzheimer’s disease (3xTg-AD) [18] because of the validity of its construction and because it largely imitates AD in humans [19]. 3xTg-AD mice have been used for studies on AD and circadian rhythmicity of locomotor activity, but mostly in a running wheel environment under light-dark cycles (LD, 12:12 h) or constant darkness (DD). Those studies showed a shortening in the circadian period in DD, reduced nocturnal but increased diurnal locomotor activity, and reduced expression of arginine-vasopressin peptide (AVP) in the SCN prior and post-Aβ pathology [20]. Ten-month-old 3xTg-AD mice showed greater amplitude in the nocturnal locomotor activity, increased body temperature, and increased food intake [21]. Also, changes in SCN clock genes were noted from 6 months old, in such a way that *Cry1* was upregulated and *Rorα* was decreased. However, changes in *Bmal1* expression were noted until 18 months (Mo), when the pathology was more advanced and could be masked by the normal aging process [22], indicating that a detrimental consequence of AD could be differentially affecting the molecular regulation of the circadian clock with age.

SCN entrainment by the LD cycle requires photic input from the RHT [23]. Changing the LD schedule to produce jet lag is a model of forced de-synchronization that allows the study of the adaptive mechanisms of the SCN to maintain coordination with the peripheral clocks [24]. To date, no study using 3xTg-AD mice has analyzed the effect of jet lag at different ages, and our could be a reference to better understand the progressive circadian alterations in AD. In this context, it has been reported that 3xTg-AD mice showed deposits of Tau protein [25,26] and β-amyloid protein [26,27] in the retina; therefore, retinal activity represents a strategy for the study of AD progression, from early stages of neuronal alteration to stages with marked neurodegeneration. Hence, the aim of this project was to better understand the progressive modifications (at 3, 8 and 13 Mo) in circadian responses related to jet lag in 3Tg-AD mice. We also explored potential coincidences between the gradual processes of neurodegeneration and retinal function.

## Materials and Methods

### Animals

All procedures were done in accordance with the international ethical guidelines of the Declaration of Helsinki, the National Institutes of Health Guide for the Care and Use of Laboratory Animals (NIH Publication No. 8023), Mexican Official Standard NOM-062-1999 and the INB-UNAM Bioethics Committee guidelines.

Homozygous triple transgenic mice for AD (3xTg-AD) were used for this study. These mice contain 3 transgenes of human origin (PS1M146V, APPSwe, and tauP301L) in a C57BL6/J background. Non-transgenic (NoTg) control mice contained non-transgenes [18]. The control mice used in this study were NoTg B6129SF2/J stock No. 101045, which the Jackson Laboratory Company recommends when 3xTg-AD B6 129-Tg (APPSwe, tauP301L) 1LfaPsen1tm1Mpm/Mmjax is used as an experimental group. Mice were kept individually in acrylic boxes in a controlled environment with light-dark cycles (LD 12:12 h; photophase 06:00–18:00 h; 300 lux) and temperature at 24+1°C. The mice were fed Rodent Lab Chow 5001, Purina Inc., and water *ad libitum*. A total of 60 male mice (30 3xTg-AD and 30 NoTg) aged 3, 8, and 13 months (n = 10 in each group) were used for the study. Each age corresponded to a stage in AD according to the establishment of pathological markers: early, intermediate, and advanced.

### Recording of locomotor activity

Free locomotor activity was individually monitored using a system of infrared sensors located on the sidewalls of acrylic cages (30×20×15 cm). Locomotor activity was individually measured as the number of interruptions of infrared beam crossings. The events were summed up and stored every 10 min using ACTIBIO software, as indicated elsewhere [28]. The recordings of locomotor activity and LD shifts (jet lag protocol) were performed inside a chamber with a controlled environment (CONVIRON, ADAPTIS 1000), a relative humidity of 40% and a photoperiod of 12 h light and 12 h dark (LD 12:12, white light LED, 300 lx) regulated by a timer. Zeitgeber time (ZT) was used as a reference; lights on = ZT0 and lights off = ZT12. The cages were cleaned once a week during the photophase and before and after DD. Locomotor activity was represented by percentile actograms using ClockLab software (Actimetrics Inc., Wilmette, IL, USA).

### Experimental protocol (Jet lag and Free Running)

Each LD or DD cycle was maintained for at least 14 days. Initial locomotor activity (INI) was recorded at LD 12:12 equivalent to the vivarium conditions (lights on 06:00 h, lights off at 18:00 h); then, the photoperiod was advanced by 6 h (ADV, lights off at 13:00 h). After 2 weeks, the photoperiod was delayed by 6 h (DEL, lights off at 18:00 h). Finally, the animals were left in DD.

### Analysis of circadian parameters of locomotor activity

Locomotor activity was averaged, analyzed and represented in double-plotted actograms. The last 5 days of each photoperiod were considered stable conditions to reduce the effect of transient cycles and were used to plot the individual average activity profile and phase angle of activity onset with lights off (Ψ). After shifting the photoperiod, the number of transient cycles to re-entrainment and acrophase of the last 5 days were analyzed using ClockLab software with the following criteria: Onset on: 9, off: 6; Offset on: 3, off: 8. The onset activity we used was a modification based on Sellix et al. [29]: The onset of stable activity in full entrainment was considered on the last days of each LD condition when no differences in day-by-day comparisons were noted (one-way ANOVA). Five days with no differences in onset were considered to calculate the phase angle of entrainment (Ψ± SEM). Transients to re-entrainment were evaluated as days required to reach a new phase with a stable Ψ (± SEM). The total average, diurnal, and nocturnal amounts of locomotor activity were also compared between groups. A modification in the method reported by Bittman [30] was used to analyze the masking effects of light: a 6 h average activity of 3 nights before the LD shift was used as a reference and compared to activity in the projected schedule on the first day after the LD shift. When LD was advanced, we used the first half (6 h) of the subjective night, and when LD was delayed, we used the second half (6 h) of the subjective night; the change was plotted in percentage differences regarding its control. The circadian period of the locomotor activity rhythm under DD conditions was calculated by the periodogram of X^2^ [31], the amplitude of Qp value was considered as the prominence of the circadian component; the acrophase calculated in ClockLab software. Significant circadian rhythm was considered when periodogram amplitude was above the significance reference (P < 0.5).

### Electroretinogram

The flash electroretinograms (fERG) of NoTg mice at 3, 8, and 13 Mo were recorded after 24 h of adaptation to darkness or constant illumination at regular photoperiod intensity. The mice were anesthetized with a mixture of 70% ketamine and 30% xylazine (1 μl/g body weight, i.p.). Then, in each eye, a drop of a solution of 0.5% tropicamide and 0.5% phenylephrine was applied for pupil dilation [32]. The cornea was kept hydrated with Hypromellose 0.5%. The body temperature was maintained with a heating pad set. A silver chloride ring electrode was placed on the cornea of each eye, 2 reference electrodes were placed subcutaneously near the eyes, and the ground electrode was placed on the base of the tail. For the recording under mesopic conditions (mixed rod and cone response), mice were kept in darkness for 12 h before the recording, which was done with the support of a red light and stimulating a 1 ms light pulse with an intensity of 0.9 log cd x s/m^2^ (PS33 Plus Photo Stimulator, GRASS Technologies, Warwick, RI). The response was also evaluated under photopic conditions, for which the mice were adapted to a 25 cd/m^2^ background light for 1 h. For both ERG conditions, the signals were amplified 2000 times by a GRASS P511AC amplifier (passband adjusted from 3 Hz to 0.3 kHz) and digitized with an A/D converter (Polyview adaptor unit, PVA-16, Grass Instruments). Sixteen successive signals were averaged, and the amplitude of wave A and wave B was determined, as well as the implied times for each latency [33].

### Data Analysis

Data were collected for experimental conditions (non-transgenic and transgenic mice) and time (3, 8 and 13 Mo) and are presented as mean ± standard error of the mean. Further, the data were compared with two-way ANOVA (to compare control and experimental groups for each age and LD conditions) and independent measures with one-way ANOVA (to test differences inside each group among LD shifts or ages); significant values were set at P < 0.05. Twenty-four hour diurnal fluctuations in locomotor activity were analyzed with ClockLab. Statistical analysis was done with GraphPad Prism 6.01 software.

## Results

### Locomotor activity

**Figure 1:**
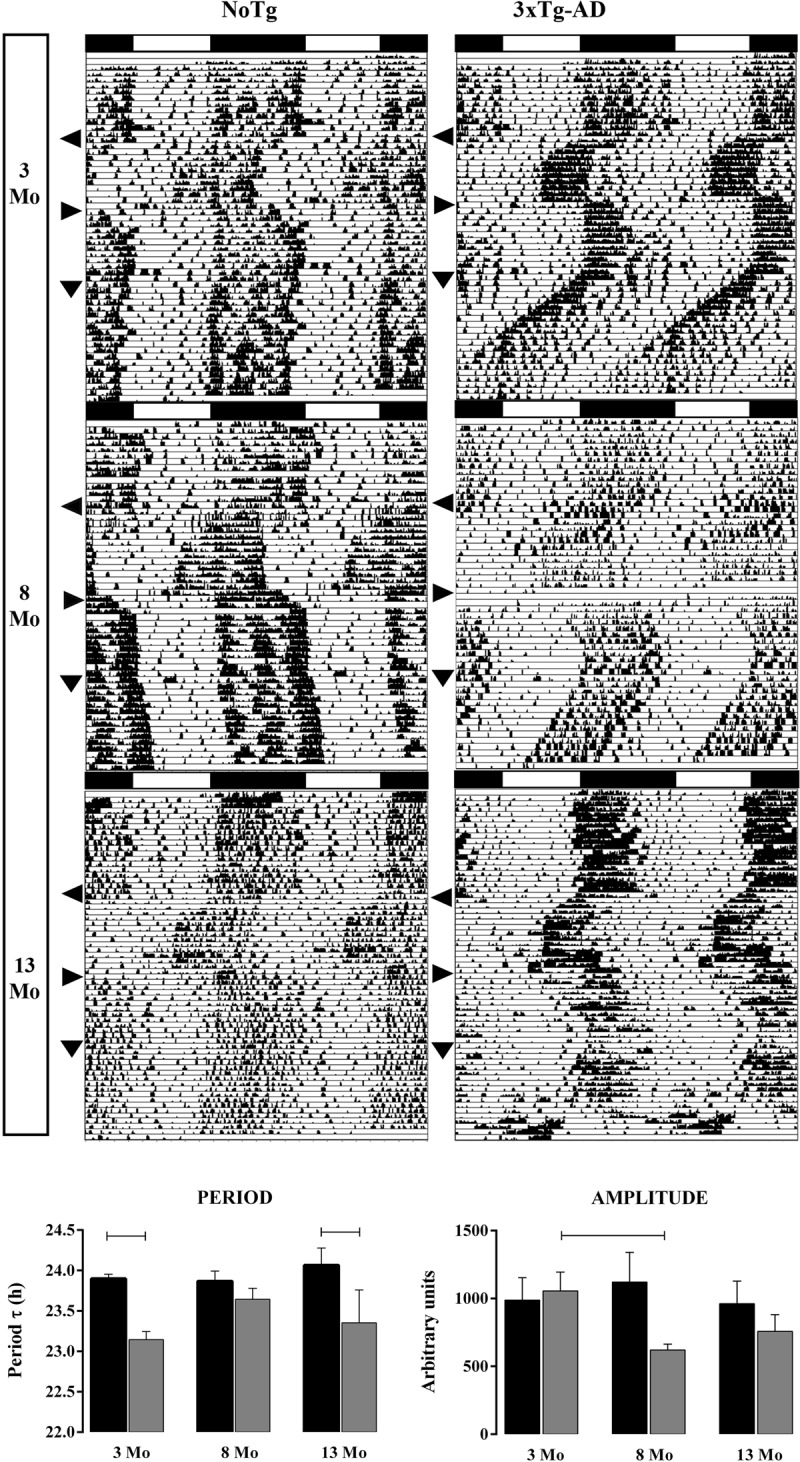
Percentile actograms of NoTg and 3xTg-AD mice at 3, 8, and 13 Mo. In each actogram, an arrowhead indicates the advance (to the left), the delay (to the right), and the start of DD (downwards). The lower panel shows the average (±SE) of free-running periods in DD (left) and amplitude (right). NoTg mice in black and 3xTg-AD mice in gray bars (two-way ANOVA with Tukey’s post hoc test, p < 0.05, n = 7–13).

***Figure 1*** shows representative actograms of the 24 h rhythm of locomotor activity of NoTg (left) and 3xTg-AD (right) mice at 3, 8, and 13 Mo and different stages of the jet lag (6 h of Advance and Delay) and DD protocols. Mice remained at least 14 days in the same condition to observe the full adaptation to each lighting condition. In all cases, there was an entraining response to the LD shift and at the end, a free-running circadian rhythm in DD. The most evident difference in 3xTg-AD mice was the shortening of the period at 3 and 13 Mo. The free-running circadian τ was assessed for all 3 ages, and significant differences were found between groups at 3 Mo (two-way ANOVA. NoTg: 23.90 ± 0.06 h versus 3xTg-AD: 23.14 ± 0.10 h; F1, 52 = 11.27, P = 0.0015) and 13 Mo (2-way ANOVA. NoTg: 24.07 ± 0.21 h versus 3xTg-AD: 23.35 ± 0.41 h; F = 1, 52 = 11.27, P = 0.0015). For the Qp amplitude, no significant differences were found between groups, except in 3xTg-AD groups (one-way ANOVA. F (2, 27) = 3.6640, p = 0.0391). In contrast, the shortening persisted at 8 Mo (lower panel), but the difference was not statistically significant.

### Analysis of the activity profiles after Photoperiod Advance and Delay

**Figure 2:**
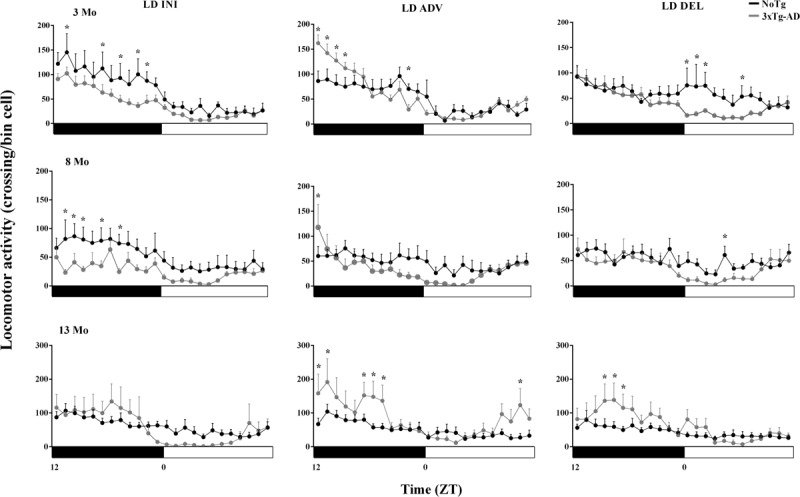
Average steady-state activity profiles per hour (±SE) of the NoTg mice (black lines, n = 26) and 3xTg-AD mice (gray lines, n = 28). Initial (top), 6h Advance (middle) and 6h Delay (bottom). The asterisk indicates differences (two-way ANOVA with Tukey’s post hoc test, p>0.05) between groups throughout the LD cycle.

The analysis of locomotor activity profiles (5 consecutive days with no difference in onset values) entrained to LD cycles is shown in ***Figure 2***. At 3 and 8 Mo, in the initial condition, 3xTg-AD mice displayed less activity than NoTg mice mainly during the night, but at 13 Mo this difference was lost (left panel). After the LD advance (middle panel), the activity of 3xTg-AD mice at 3 Mo increased almost two-fold immediately after lights off and gradually decreased throughout the night. After the LD delay (right panel), the activity between groups (3 and 8 Mo) was similar in the dark hours and gradually decreased in 3xTg-AD mice after lights on. At 13 Mo, 3xTg-AD mice showed great variability in average nighttime activity, with a tendency to be both more active and less active during the day than NoTg mice. The main changes in the activity profiles were the increase in 3xTg-AD activity after the advance of the schedule at 3 and 13 Mo, and the significant increase in nighttime activity at 13 Mo in INI, ADV and DEL. However, in the ADV protocol, this increase was also seen during the second half of the light period.

The comparisons of daytime and nighttime activity and the global average during the last 5 days of each condition are shown in ***Table 1***. Intragroup differences were tested by One-Way ANOVA, while intergroup differences were tested with Two-way ANOVA either between age or light conditions. In the INI protocol, NoTg mice did not show any changes in global, diurnal or nocturnal activity regardless of age. However, 3xTg-AD mice showed a significant increase (123%) in global activity at 8 Mo to 13 Mo and in nocturnal activity (153%) at 13 Mo.

**Table 1:** Statistical Analysis of chronobiological experiments in 3xTg-AD mice under jet lag protocol. On top, the average (± SE) on Total, Day and Night locomotor activity (movements/bin cell) at 3, 8 and 13 Mo, between no transgenic (NoTg, n = sample size) and triple transgenic mice for Alzheimer disease (3xTg-AD). Below are shown the average (± SE) acrophases in Zeitgeber Time (ZT). Statistical differences inside each group (One way ANOVA) are indicated between data with same symbols. Differences between NoTg and 3xTgAD mice (between groups; Two-way ANOVA) are indicated with same letter.


	AVERAGE ACTIVITY (MOVEMENTS/10 MIN)			

	INITIAL	TOTAL	NoTg(n = 7–10)	3xTg-AD(n = 7–13)

**3 Mo**			65.40 ± 14.20^c^	37.94 ± 6.10^c^

**8 Mo**			37.90 ± 6.22	25.70 ± 5.16^#^

**13 Mo**			39.90 ± 8.50	57.25 ± 13.60^#^

		**DAY**		

**3 Mo**			28.61 ± 8.84	17.8 ± 3.40

**8 Mo**			19.20 ± 3.42	14.40 ± 2.23

**13 Mo**			20.90 ± 4.64	20.90 ± 8.30

		**NIGHT**		

**3 Mo**			102.20 ± 26.20	59.43 ± 13.61

**8 Mo**			56.60 ± 10.20	36.95 ± 8.24^&^

**13 Mo**			58.91 ± 15.14	93.62 ± 22.46^&^

	**ADVANCE**	**TOTAL**	**NoTg(n = 7–10)**	**3xTg-AD(n = 7–13)**

**3 Mo**			52.20 ± 10.00	52.73 ± 5.16

**8 Mo**			29.13 ± 5.70 ^e^	30.48 ± 9.40*

**13 Mo**			43.05 ± 10.60^a,e^	82.70 ± 19.74^a^,*

		**DAY**		

**3 Mo**			26.80 ± 9.64	21.33 ± 2.14^†^

**8 Mo**			17.75 ± 4.17	19.00 ± 5.45^‡^

**13 Mo**			21.61 ± 9.40^b^	51.30 ± 14.54^b,†,‡^

		**NIGHT**		

**3 Mo**			77.61 ± 13.50	84.12 + 9.80

**8 Mo**			40.51 ± 7.82^f^	42.00 ± 14.50@

**13 Mo**			64.50 + 13.43^f^	114.12 ± 28.32@

	**DELAY**	**TOTAL**	**NoTg(n = 8–11)**	**3xTg-AD(n = 7–13)**

**3 Mo**			58.22 ± 10.54^d^	37.7 ± 6.65^d^

**8 Mo**			47.07 ± 6.00	33.56 ± 8.94

**13 Mo**			43.55 ± 9.90	62.99 ±18.10

		**DAY**		

**3 Mo**			49.65 ± 19.34	21.10 ± 3.73

**8 Mo**			37.66 ± 6.21	21.10 ± 7.13

**13 Mo**			26.25 ± 8.20	33.10 ± 13.43

		**NIGHT**		

**3 Mo**			66.80 ± 12.10	54.33 ± 12.10

**8 Mo**			56.50 ± 9.80	46.10 ± 11.80

**13 Mo**			60.90 ± 14.40	92.90 ± 30.43

	**ACROPHASES (ZT)**			

	**INITIAL**		**NoTg(n = 8–11)**	**3xTg-AD(n = 7–13)**

**3 Mo**			17.61 ± 0.23	16.24 ± 0.26

**8 Mo**			17.19 ± 0.49	16.12 ± 0.62

**13 Mo**			16.53 ± 0.53	16.70 ± 0.43

	**ADVANCE**			

**3 Mo**			17.80 ± 0.49^g^	15.40 ± 0.20^g^

**8 Mo**			17.30 ± 0.46^h^	15.38 ± 0.36^h^

**13 Mo**			17.02 ± 0.37^i^	15.22 ± 0.64^i^

	**DELAY**			

**3 Mo**			16.60 ± 0.63	15.86 ± 0.32

**8 Mo**			16.25 ± 0.53	15.67 ± 0.35

**13 Mo**			14.40 ± 0.73	15.64 ± 0.82


In the ADV protocol, NoTg mice did not show any changes in global, diurnal or nocturnal activity regardless of age. 3xTg-AD mice displayed a significant increase in global activity (171%) at 8 and 13 Mo. Diurnal activity increased 140% at 13 Mo compared to 3 Mo, and 170% between 8 and 13 Mo. Nocturnal activity increased 171% from 8 Mo to 13 Mo.

In the DEL protocol, NoTg and 3xTg-AD mice did not show changes in global, diurnal or nocturnal activity regardless of age.

The differences between NoTg and 3xTg-AD mice were in INI protocol at 3 Mo where there was a 42% decrease in global activity in the transgenic mice. In the ADV protocol, 3xTg-AD mice exhibited an increase in global and diurnal activity (92% and 137%, respectively) at 13 Mo. In the DEL protocol, the global activity of 3xTg-AD mice decreased by 35% with respect to their control.

The acrophases in the ADV protocol were different for all 3 ages.

**Supplementary Figure 1:**
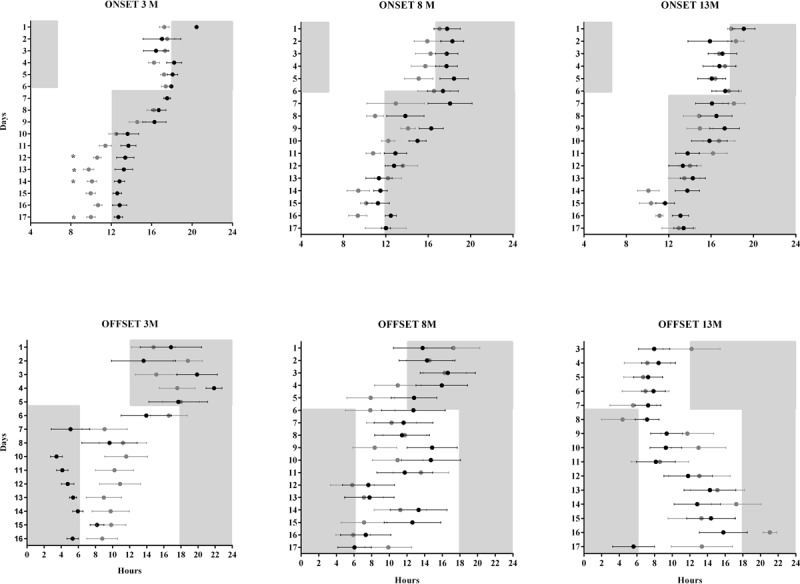
Average (±SE) of the onset (upper panels) and offset of locomotor activity (lower panels). The Onset is shown just for advances in LD schedules, while the offset is shown just for delays, to exclude the masking effect of light. Three ages are compared: 3, 8 and 13 Mo, respectively. Grey circles correspond to data from 3xTgAD mice, and black circles to its control group. Significant differences between groups are noted with a star (2way ANOVA, P0.05).

A new evaluation on the progression of activity onset and offset was done to analyze re-entrainment, as shown in ***Supplementary Figure 1***. No significant differences were observed in the number of days required to re-entrain to LD advances, when onset was used as a reference. The number of days to re-entrain to LD advances was defined as those needed to shift the onset of activity to the next stable calculated Ψ value (***Figure 4*** and ***Supplementary Figure 1***, onset). Transients to re-entrain LD delays were not considered due to the masking effect on activity onset and the variability in activity offset (***Supplementary Figure 1***, offset).

### The masking effect of light

**Figure 3:**
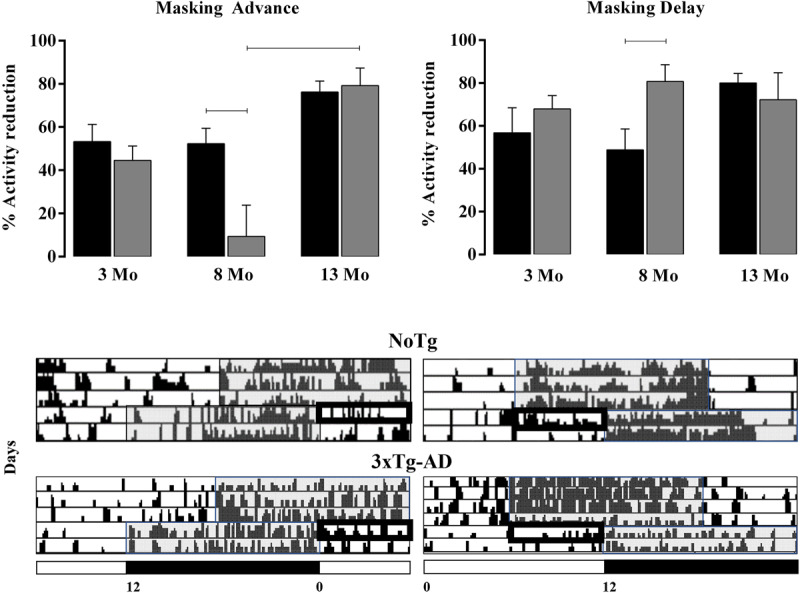
Average (±SE) of the phase angle (Y, min) between the onset of activity and lights-off. NoTg (black, n = 26) and 3xTg-AD (gray, n = 28) mice at initial (Ini), Advance (Adv) and Delay (Del) in LD conditions. The differences are indicated with brackets (two-way ANOVA per age, Tukey’s post hoc test, p < 0.05).

The negative masking of locomotor activity by light is summarized in ***Figure 3***. After advances (C), light in the second half of the subjective night reduced the activity by 50% in both NoTg and 3xTg-AD mice at 3 Mo. Then, at 8 Mo, 3xTg-AD mice showed a minimum change (9.4%), and 4 mice showed positive masking (more active than the reference; negative values are not plotted). At 13 Mo, negative masking was nearly 80% in both groups. After LD Delays, when light was present in the first half of the subjective night, negative masking at 8 Mo was almost 50% in NoTg and 80% in 3xTg-AD mice.

**Figure 4:**
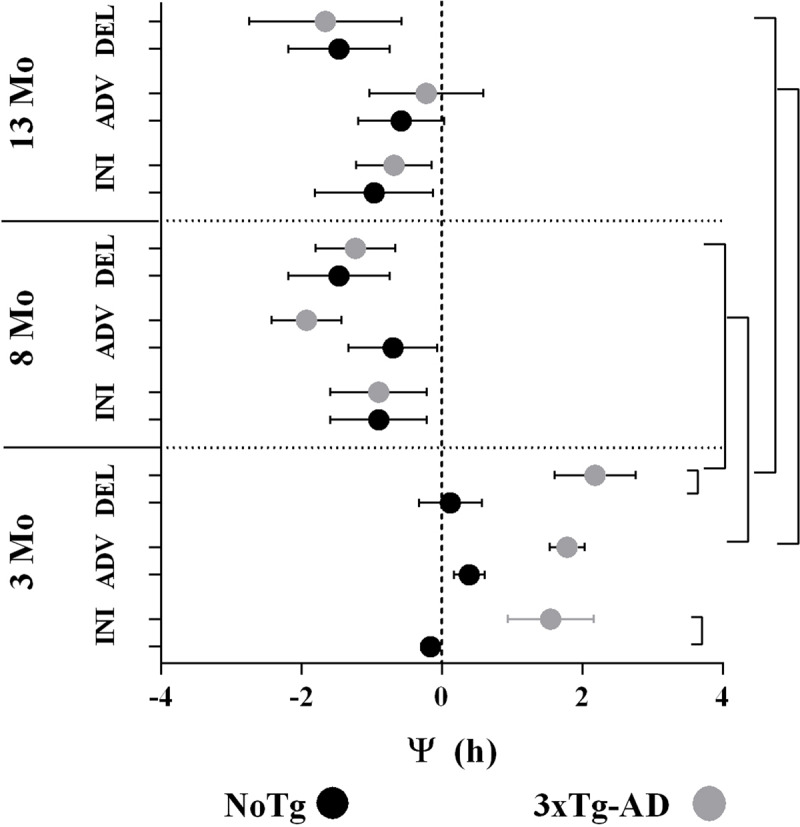
Average (±SE) analysis of the masking effect of light (% reduction). NoTg mice are represented in black and 3xTg-AD mice in gray bars. Significant differences among groups are indicated with brackets (two-way ANOVA per age, Tukey’s post hoc test, p < 0.05 test, n = 6–10 per group). Below, fragments of representative actograms.

The phase angle (Y, h) between the onset of activity and lights-off (indicated as 0 in ***Figure 4***) was not different between NoTg mice regardless of age. However, Ψ was notably advanced in 3xTg-AD mice at 3 Mo. In the INI protocol, a significant difference was observed between groups (two-way ANOVA. NoTg: -0.16 ± 0.13; 3xTg-AD: 1.55 ± 0.61. F = 1,198 = 18.76; p = < 0.0001), as well as in the DEL protocol (NoTg: 0.12 ± 0.44; 3xTg-AD: 2.18 ± 0.57. F = 1,198 = 18.76; p = < 0.0001). 3xTg-AD mice exhibited differences in Ψ at 3 Mo (1.78 ± 0.24) in comparison with 8 Mo (1.92 ± 0.49) and 13 Mo (-0.22 ± 0.81) (two-way ANOVA F = 2, 189 = 8.926. P = 0.0002). In the DEL protocol, differences were identified at 3 Mo (2.18 ± 0.57) in comparison with 8 Mo (–1.229 ± 0.56) and 13 Mo (–1.65 ± 1.08) (two-way ANOVA F (2, 161) = 10.08. P < 0.0001).

### Electroretinogram analysis

**Figure 5:**
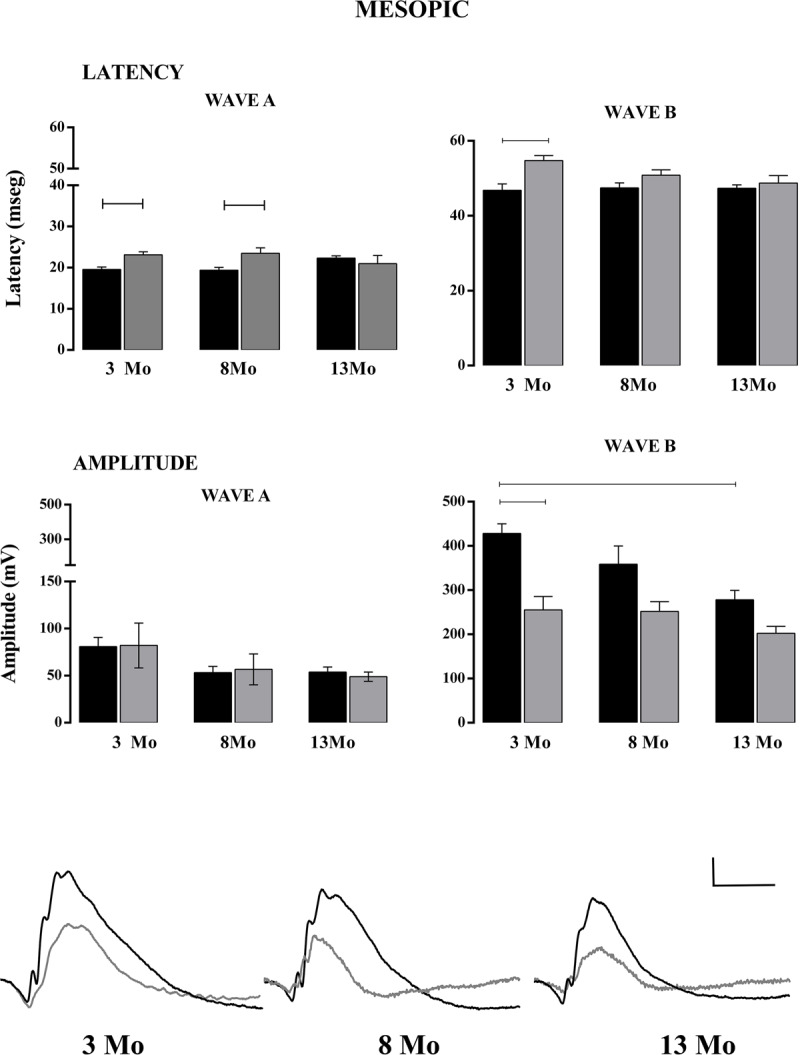
Latency and amplitude analysis under mesopic conditions. In the upper panel, the latency of waves A and B. In the lower panel, the amplitude of waves A and B. Representative traces of ERG at 3 Mo, 8 Mo and 13 Mo. NoTg animals are depicted in black and 3xTg-AD animals in gray. Two-way ANOVA per age, Tukey’s post hoc test, p < 0.05 test, n = ±8. Calibration bar: 100 μV/100 msec.

**Figure 6:**
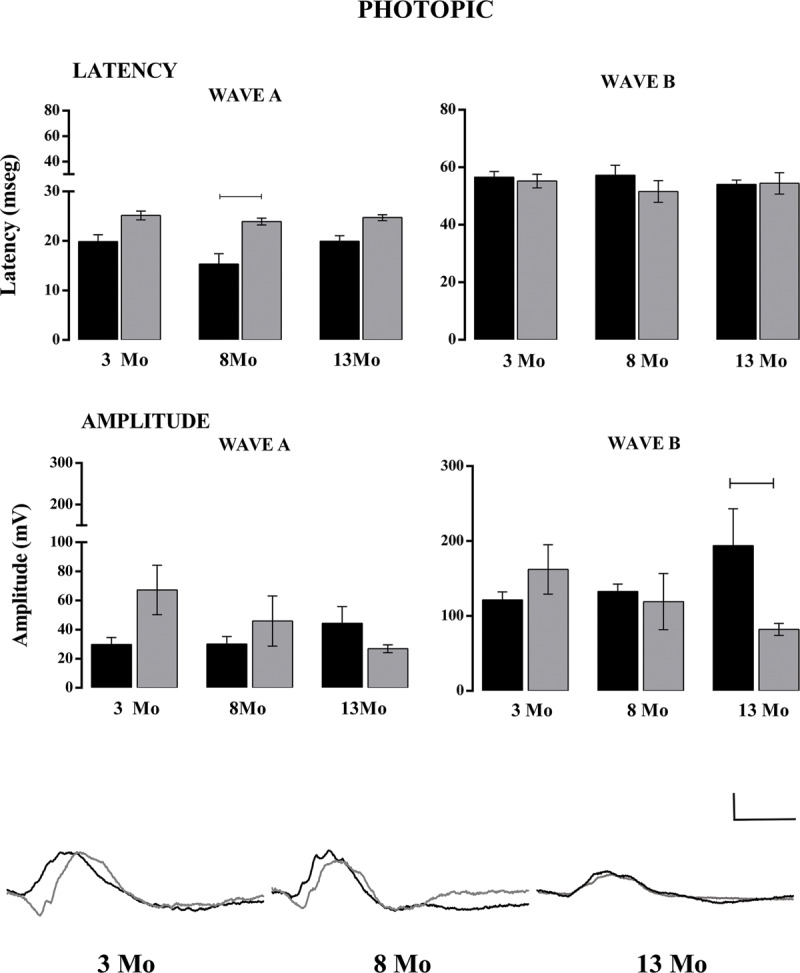
Latency and amplitude analysis under mesopic conditions. In the upper panel, the latency of waves A and B. In the lower panel, the amplitude of waves A and B. Representative traces of ERG at 3 Mo, 8 Mo and 13 Mo. NoTg animals are depicted in black and 3xTg-AD animals in gray. Two-way ANOVA per age, Tukey’s post hoc test, p < 0.05 test, n = ±8. Calibration bar: 100 μV/100 msec.

We explored if the functional retinal response was progressively affected in the 3xTg-AD mice at 3, 8 and 13 Mo, which correspond to early, intermediate and advanced states of neurodegeneration. ***Figure 5*** shows the average latency and amplitude (±SE) of wave A in both NoTg (black) and 3xTg-AD (gray) mice. Under mesopic conditions, the next significant differences were found (upper, left panel) at 3 Mo (two-way ANOVA. NoTg: 19.02 ± 0.78, n = 22; 3xTg-AD: 23.11 ± 0.72, n = 9. F = 1, 69 = 15.01, P = 0.0002) and 8 Mo (two-way ANOVA. NoTg: 18.92 ± 0.80, n = 14; 3xTg-AD: 23.50 ± 1.31, n = 7. F = 1, 69 = 15.01, P = 0.0002). The B-wave latency was greater in 3xTg-AD mice at 3 Mo (upper right panel) (two-way ANOVA 46.79 ± 1.75, n = 22; 3xTg-AD: 54.72 ± 1.31, n = 11. F = 1, 76 = 9.05, P = 0.0035). Moreover, no differences in the A wave amplitude were found between both groups and the 3 ages (middle, left panel). However, B-wave amplitude decreased significantly with age in NoTg mice comparing 3 Mo and 13 Mo (two-way ANOVA. 3 Mo: 428.30 ± 21.42, n = 19; 13 Mo: 278.12 ± 21.09, n = 11. F = 2, 66 = 6.86, P = 0.0020), whereas it remained modest from 3 Mo to 13 Mo in 3xTg-AD mice (middle right panel). There were significant differences between NoTg and 3xTg-AD at 3 Mo (two-way ANOVA. NoTg: 428.30 ± 21.42, n = 19; 3xTg-AD: 255.31 ± 30.45, n = 9. F = 1, 66 = 25.85. P < 0.0001). Representative traces of the average temporal course of the electroretinogram response recorded under mesopic conditions for all ages are shown at the bottom (n = 5–10). Under photopic conditions, A-wave latency in 3xTg-AD mice increased at the 3 ages (***Figure 6***), although this increase was significant only at 8 Mo (upper left panel; two-way ANOVA. NoTg: 15.36 ± 2.08, n = 11; 3xTg-AD: 23.92 ± 0.69, n = 5), possibly because of the decrease in the latency of NoTg mice. There was no significant difference in A- and B-wave amplitudes, but the B-wave was decreased in 3xTg-AD mice at 13 Mo with respect to the control (two-way ANOVA. NoTg: 193.79 ± 49.36, n = 15; 3xTg-AD: 82.02 ± 8.01, n = 15).

## Discussion

To our knowledge, the present study is the first to characterize fine-tuned chronobiological parameters in 3xTg-AD mice under conditions of free movement and not using a running wheel. This difference is relevant since wheel-running activity promotes metabolic and physiological adaptations that influence the free-running period as well as in the strength of the rhythms [34,35]. The neurodegenerative process displayed in 3xTg-AD mice is complex, and histopathological markers of AD (β-amyloid plaques and hyperphosphorylated tau protein) were expressed in varying degrees across different brain zones [18]. In addition, functional and neuronal abnormalities are not always directly correlated [36]. In the case of the SCN, we did not detect the presence of β-amyloid plaques in 3xTg-AD mice (data not shown).

### Circadian disturbances in 3xTg-AD mice

The results indicated that 3xTg-AD mice at 13 Mo increased total locomotor activity and photophase activity after LD advance. In the scotophase, the trend of increased activity was maintained but did not reach statistical significance. This result contrasts with studies that have recorded locomotor activity using the running wheel [20,37]. Therefore, the recording method might influence these discrepancies. Studies involving the running wheel reflect the interaction between behavioral and motivational factors that can induce obsessive and addictive behaviors [38]. Leise et al. [35] in 2013 found that the access to a running wheel alleviated some age-related changes in the circadian system like changes in the period, re-entrainment capacity after a shift in the LD cycle and firing rate in explants of the SCN. The differences with the study by Sterniczuk et al. [20] are possibly due to the use of a wheel and their choice of putting together a pool of mice before and after the establishment of histopathological markers. However, we believe that an environment without a running wheel allows researchers to observe spontaneous motor activity without the motivational influence of the wheel and reduces the effect of physical exercise that delays the development of AD by increasing neuronal and vascular plasticity in areas affected by AD [39,40].

Interestingly, 3 Mo 3xTg-AD mice showed increased activity after LD advance at the onset of darkness but not after LD delays. This behavior had not been observed before in this model for AD and could imply a different regulation from the SCN to other brain centers that regulate activity, including the dorsomedial hypothalamus [41]. Within the SCN, there is a gradient in which the neurons of the ventral region show faster phase-shifting than those of the dorsal region upon a 6 h advance [42]. The speed at which clock genes change to a new schedule differs in advances and delays [43]. 3xTg-AD mice also show reduced vasopressinegic and vipergic cell populations in the SCN [20], which may contribute to a faster re-entrainment after the LD shift. It has been reported that mice lacking vasopressin receptors V1a-/-V1b-/- are slightly responsive to jet lag [43]. Further studies in 3xTg-AD mice are necessary to elucidate the relationship between AD progression and atypical circadian responses to entrainment.

Hypothalamic areas that integrate behavior and metabolism deteriorate in AD [44]. In previous reports, the enhanced activity related to sundowning has been related to cellular adaptation that takes place at the beginning of the resting phase [8]. In the present study, we observed that the enhanced activity was facilitated during the advance of the photoperiod and appeared at the beginning of the activity phase at 3 Mo. At 13 Mo, the activity increased in both the photophase and the scotophase. A putative sundowning should have showed a significant increase in activity at the end of the subjective night, but it was not observed in this work.

Furthermore, we showed in 3 Mo 3xTg-AD mice a shortening of the circadian period in DD, supporting reports by Sterniczuk et al. [20] and Wu et al. [6] indicating that period shortening in organisms below 6 months of age is possible. However, we did not detect the presence of β-amyloid in the SCN at any of the ages studied (data not shown), when the presence of β-amyloid plaques in several cerebral zones has been observed [18]. The shorter period at early age suggests a change in the circadian clock machinery in 3xTg-AD mice that is not directly associated with the presence of traditional histopathological markers of AD. Stopa et al. [45] found neurofibrillary tangles in the SCN of patients with advanced dementia. It has been noted in other studies that AD is associated with changes in the activity of phosphorylating enzymes, such as casein kinase I [46], and that modulation of the activity of casein kinase I δ/ɛ changes the free-running period and advances or delays in entrainment to LD [47]. Therefore, phosphorylation of molecular components of the circadian clock is linked to the implementation of a short free-running period and advanced onset of activity. Also, the enzyme GSK3β has been related to molecular clock functioning and AD; it phosphorylates at least 5 clock proteins and regulates the period of the PER protein [48]. Moreover, GSK3β promotes neurofibrillary tangles that interrupt the correct assembly of the neuronal cytoskeleton and cause synaptic loss [49].

The transient cycles, masking, and phase angle are indicators of the circadian timing system’s ability to resynchronize during a jet lag protocol. These parameters are altered with age, since older individuals require more transient cycles to reach a stable phase [5,29]. We did not find differences in transients to LD advance even in the youngest mice (***Supplementary Figure 1***) with a more constant Ψ. The assessment of differences to re-entrain to LD delays is masked when using onset (***Figure 4***), and a high variability in offset of activity reduce to observe possible differences. It was previously noted that no differences in photic phase-shifting existed between NoTg and 3xTg-AD mice [20]. Another effect of the short free-running period is the advance in acrophase and activity onset in young 3xTg-AD mice.

The direct retinal connection to the SCN and intergeniculate leaflet (IGL), which also receives direct input from the retina and connects with the SCN, is involved in the regulation of synchronization and masking [50]. Negative masking is the suppression of locomotor activity in the presence of light. We observed that the masking effect of light is different when it is elicited in the first or second half of the night, indicating that masking also exerts circadian regulation and that diverse neural mechanisms participate in such response [51]. The differences observed in the present work suggest that further studies are required to understand why masking is different in 3xTg-AD mice. A first approach using ERG indicates a deficiency in mesopic, but not photopic, B-wave amplitude. Retinal degeneration reduces negative masking in bright light and may facilitate positive masking in dim light [52].

In our results, there was an immediate increase in locomotor activity in 3xTg-AD mice at 8 Mo in the LD advance (***Figure 4C***), indicating positive masking. This finding suggests a reduced sensing of light; however, in the amplitude of wave A, no significant differences were observed, indicating that there is no alteration in photoreception and that the light input reaching the external retina is being correctly integrated. Although the photoreceptors performed properly, our data suggest that some relays in the internal retina could have undergone a functional change. Further studies on ipRGCs, which play a predominant role in this masking phenomenon, are needed [53]. The 3xTg-AD mice showed fewer transient cycles to reach a stable phase after LD advance, which is consistent with a short free-running period of the circadian rhythm of locomotor activity. The onset of activity in young animals is also affected by the intrinsic period and ZT [23]. However, even though the free-running period remains short with age, the phase angle at lights-off becomes delayed in older mice (13 Mo), indicating that other deteriorating conditions could occur between the pacemaker and the output of the clock.

### Electroretinogram

The fERG recording in mesopic conditions (darkness) involves the response of retinal cells within the outer layer, so it is not possible to separate the response from the ipRGC. In the retinal outer layer, the response of photoreceptors (mainly rods, but also cones) (wave A), bipolar cells, and Müller cells (wave B) takes place. Under photopic conditions, the response is given only by cones [54]. However, this type of ERG is useful to identify alterations in the functionality of extracerebral neural tissue, which supports the premise that the retina is a “window” to study the brain. In this context, ERG is a tool to observe early events in a non-invasive protocol. We showed that in 3xTg-AD mice, the decrease in B-wave amplitude under mesopic conditions occurred in early stages of AD pathology (3 Mo), whereas in NoTg mice, the reduction happened progressively with age. These results could be related to a study by Grimaldi et al. [26], who found amyloid and tau protein deposits, ganglion cell degeneration, astrogliosis and microglial activation in the retina of 3xTg-AD mice (5–20 weeks of age).

## Conclusions

Based on a 24 h free locomotor activity analysis, without running-wheel devices, we demonstrated that the circadian rhythm of locomotor activity in young 3xTg-AD mice was different under a jet lag protocol and DD at an age previous to the detection of classical histopathological markers of AD (3 Mo). Moreover, 3xTg-AD mice showed reduced retinal function starting at 3 Mo. Entrainment to LD did not show deficits, indicating that the circadian system has resilience mechanisms that make it resistant to the neurodegenerative process characteristic of these transgenic mice. Our results suggest that this jet lag protocol (basec on actigraphy) and fERG can be added to the extensive battery of tests available for early and non-invasive detection of AD.

A note of caution is pertinent: It cannot be ruled out that the chronobiological differences detected in this study between NoTg and 3xTg-AD mice could be due to a genetic drift or another chromosomal alteration resulting from the multiple molecular manipulations to construct the triple transgenic mice.

## Additional File

The additional file for this article can be found as follows:

10.5334/jcr.214.s1 “Changes in 24 h rhythmicity of spontaneous locomotor activity in the triple transgenic mouse for Alzheimer’s disease (3xTg-AD) in a jet lag protocol: Correlations with retinal sensitivity”. Chronobiological study to characterize the progressive alteration in spontaneous daily rhythmicity in an experimental model of Alzheimer disease.
